# Internet-delivered cognitive-behavioural therapy for concerned significant others of people with problem gambling: study protocol for a randomised wait-list controlled trial

**DOI:** 10.1136/bmjopen-2015-008724

**Published:** 2015-12-09

**Authors:** Kristoffer Magnusson, Anders Nilsson, Clara Hellner Gumpert, Gerhard Andersson, Per Carlbring

**Affiliations:** 1 Department of Clinical Neuroscience, Stockholm Center for Psychiatry Research and Education, Karolinska Institutet, Stockholm, Sweden; 2 Department of Behavioral Sciences and Learning, Linköping University, Linköping, Sweden; 3 Department of Psychology, Stockholm University, Stockholm, Sweden

**Keywords:** concerned significant other, pathological gambling, cognitive behavior therapy, randomized controlled trial

## Abstract

**Introduction:**

About 2.3% of the adult population in Sweden are considered to suffer from problem gambling, and it is estimated that only 5% of those seek treatment. Problem gambling can have devastating effects on the economy, health and relationship, both for the individual who gambles and their concerned significant other (CSO). No empirically supported treatment exists for the CSOs of people with problem gambling. Consequently, the aim of this study is to develop and evaluate a programme aimed at CSOs of treatment-refusing problem gamblers. The programme will be based on principles from cognitive behavioural therapy (CBT) and motivational interviewing. To benefit as many CSOs as possible, the programme will be delivered via the internet with therapist support via encrypted email and short weekly conversations via telephone.

**Methods and analysis:**

This will be a randomised wait-list controlled internet-delivered treatment trial. A CBT programme for the CSOs of people with problem gambling will be developed and evaluated. The participants will work through nine modules over 10 weeks in a secure online environment, and receive support via secure emails and over the telephone. A total of 150 CSOs over 18 years of age will be included. Measures will be taken at baseline and at 3, 6 and 12 months. Primary outcomes concern gambling-related harm. Secondary outcomes include the treatment entry of the individual who gambles, the CSO's levels of depression, anxiety, as well as relationship satisfaction and quality of life.

**Ethics and dissemination:**

The protocol has been approved by the regional ethics board of Stockholm, Sweden. This study will add to the body of knowledge on how to protect CSOs from gambling-related harm, and how to motivate treatment-refusing individuals to seek professional help for problem gambling.

**Trial registration number:**

NCT02250586.

## Introduction

An estimated 70% of the Swedish population aged 16–84 years participate in gambling.^1^ Most experience no negative consequences, but for a small group of people gambling is problematic. The most recent national survey estimated that around 2.3%^1^ of the adult population suffer from problem gambling. Consequently, their gambling behaviour can have devastating effects on both their own and their concerned significant others’ (CSOs’) economic status, health and relationships. A large proportion (18%) of the adult Swedish population see themselves as CSOs of people with problem gambling.^2^ Moreover, the Swedish National Institute of Public Health has estimated that approximately 260 000 (∼3%) individuals cohabitate with an individual who gambles problematically, and among them 76 000 are children.^1^

The effects of problem gambling on the CSOs have been well documented in the literature.^3–6^ Problem gambling causes enormous financial problems for the affected family, such as debts, losses of property, loans that are overdue, maxed credit cards and being chased by creditors.^7^ As a result of these consequences, some CSOs report feeling depressed, a low quality of life and some even attempt suicide.^8^ ^9^ Other CSOs experience considerable anger and anxiety as a result of problem gambling.^4^ ^10^ CSOs also report several stress-related problems, for example, headaches, bowel problems and sleep disturbances.^11^ ^12^ The CSO's relation to the individual who gambles can also be severely affected, and many CSOs report escalating conflicts in the home, dissipation of trust and disturbed relationships with family and friends.^3^ ^4^ ^9^ ^13^ In a representative sample in Norway, Wenzel *et al*^14^ found that 63% of the CSOs reported that problem gambling had worsened the family's financial situation, and 65% reported that it had led to conflicts in the family. Many CSOs report that they are often left feeling isolated and unsupported.^15^

In Sweden, it has been estimated that only about 5% of the people with problem gambling seek professional help.^1^ Numerous researchers have suggested that CSOs can play a key role in getting these people with problem gambling to enter treatment, and they have highlighted the need to better equip CSOs to cope with problem gambling.^7^ ^13^ ^16–23^ Even though financial concerns are often the main reason that gamblers seek help,^24^ many individuals with a gambling problem report concerns for CSOs as an important reason for entering treatment.^18^ ^25^ Additionally, as many as 50% of people with problem gambling report that they rely on informal help provided by their CSO to overcome their gambling problem.^16^

Research on support programmes aimed at CSOs of people suffering from addiction has shown promising results in getting the treatment-refusing individual into treatment. The approach with the strongest empirical support is the *community reinforcement and family training (CRAFT)*.^26–28^ The CRAFT approach has been modified and tested with CSOs of people with problem gambling in two studies.^20^ ^29^ Both studies used a self-help workbook to deliver the training, and found that the programme had a significant effect on the number of days gambling, CSOs’ programme satisfaction and experiences of having their needs met. However, no differences were found between the CRAFT approach and the control group on rates of treatment engagement.

Few studies have evaluated interventions that focus on working with CSOs of people with problem gambling in their own right. In 2006, Rychtarik and McGillicuddy performed a preliminary evaluation of a coping skills training programme, aimed at CSOs with a partner that gambled problematically. They found a large reduction in symptoms of depression and anxiety in the coping skills training group relative to a wait-list control. However, they found no differences between groups on partner gambling or treatment entry. These findings should be considered highly preliminary since the study involved just 23 participants.

In 2013, The Swedish National Helpline received 600 calls (31% of total) from CSOs.^31^ Research has shown that most CSOs typically turn to self-help, online or telephone support before seeking professional help.^18^ Rodda *et al*^32^ looked at reasons why CSOs chose web-based counselling in Australia, and found that ease of access, privacy and anonymity were the main reasons. Another study on the same service^33^ found that the large majority of CSOs accessing web-based counselling reported emotional distress and impacts on relationship, social life and finances due to problem gambling. There is also evidence that shame and stigma are the main barriers for CSOs in seeking help;^18^ ^34^ ^35^ therefore, it is possible that an internet-delivered treatment could seem attractive to these CSOs. Cognitive behavioural therapy (CBT) has been readily adapted and evaluated over the internet. These internet-delivered CBT interventions have often achieved treatment effects that are comparable to face-to-face therapy in several studies.^36–38^ Moreover, internet-delivered CBT has also been efficaciously implemented with problem gamblers.^39^

### Aims and hypotheses

Earlier studies have found limited success in helping CSOs deal with problem gambling. Protecting the CSO from gambling-related harm can be achieved partly by motivating the individual who gambles to enter treatment, thus hopefully ending problem gambling, and partly by focusing on the CSO’s needs in their own right. Since the available support for CSOs is scarce in Sweden, the aim of this study is to develop and evaluate an internet-delivered CBT programme for CSOs of people with problem gambling. The programme will be inspired by CRAFT but can rightfully be seen as a CBT programme—utilising standard CBT techniques. Thus, this programme is referred to as CBT for CSOs of people with problem gambling (CBT-CSO).

The aim of this study will be to investigate the effects and feasibility of an internet-delivered CBT-CSO programme on (1) gambling-related harm both for the CSO and the individual who gambles, (2) treatment-seeking rate among the people with problem gambling, and (3) relationship functioning and mental health of the CSOs. It is hypothesised that: (1) the CBT-CSO programme will lead to a reduction in gambling-related harm, and a greater treatment-seeking rate, (2) the CBT-CSO programme will reduce the CSO's anxiety and depressive feelings, (3) the CBT-CSO programme will decrease the amount of time and money spent on gambling by the individual who gambles, (4) the CBT-CSO programme will increase the CSO's relationship satisfaction with the individual with problem gambling.

The programme will be compared to a wait-list control condition. This choice of comparator is justified, since not much is known about the efficacy and feasibility of these types of programmes in this population.

## Methodology

The study will be a randomised controlled trial with two arms: (1) the CBT-CSO programme and (2) a wait-list control. The wait-list group will be offered the CBT-CSO programme after 10 weeks.

### Study population and recruitment

Participants will be recruited nationwide through the Swedish National Gambling Helpline and via media and internet advertisements. Advertisements will be publicised nationwide to the general population in newspapers and on Facebook. Targeted advertisements will be published via Google Adwords. Volunteers will sign up to the study via a public website. After signing up, they will be invited to answer a survey of screening questions and the baseline assessment. If they are eligible, they will be invited to a short telephone interview with one of the study's counsellors. During this interview, the volunteers are informed about the study and get the chance to ask questions. If they agree to participate in the study, they are asked to send in written informed consent via mail. After the consent is received, treatment allocation is performed, and the participant is contacted within the treatment platform.

#### Eligibility criteria

For brevity, we will refer to the participant's related party which gambles as the identified patient (IP).

*Inclusion*: (1) The CSO and the IP are at least 18 years old, (2) the CSO is a parent, child, sibling, friend or partner of the IP. (3) The CSO must have had a relationship with the IP for at least 3 months. (4) Neither the CSO nor the IP has had any treatment in the past 3 months (ie, related to gambling). (5) The IP is currently refusing to start treatment for gambling problems. (6) The CSO is able to read and answer questions in Swedish, and is willing to have phone contact with a counsellor each week. (7) The IP is rated by the CSO as having gambling problems (score 8 or greater) on the Problem Gambling Severity Index (PGSI).^40^ (7) CSOs on psychotropic medication must have been on a stable dose for at least 3 months. *Exclusion*: (1) Presence of a current psychotic or bipolar disorder in the CSO or IP. (2) CSO meets PGSI criteria (8 or greater) for ongoing problem gambling.

### Counsellors

The study's counsellors will be at least a master's level clinical psychology students on their last semester, or experienced staff from the National Helpline who are trained in motivational interviewing (MI).^41^ They will assist the CSOs via encrypted emails and scheduled weekly telephone calls. The length of the calls will be a maximum of 15 minutes per week. The purpose of these calls is to provide positive feedback and answer questions that the CSO might have regarding the content of the modules. In addition to the telephone calls, the counsellors also provide written feedback once a week. They will also send short messages to reinforce the participants’ efforts. The amount of time spent on sending emails is limited to 15 min per week. The counsellors will also try to contact participants who are not responsive both via email and telephone, to see that there are no technical difficulties or other problems. The counsellors will receive training in the study manual and weekly supervision by an experienced CBT therapist.^42^

### Blinding

Neither participants nor counsellors will be blinded to treatment allocation. Baseline assessment occurs prior to randomisation, and follow-up assessment will be self-reported via the internet.

### Trial arms

#### CBT-CSO

The CBT-CSO programme will be based on concepts from CBT, integrative behavioural couples therapy (IBCT)^43^ and MI.^44^ CBT-CSO will be similar to the CRAFT approach in many ways, since both approaches utilise generic CBT techniques, such as psychoeducation, functional analysis and positive reinforcement. Both methods are also targeted specifically at CSOs alone, where the person with the drinking or gambling problem does not participate in the treatment. However, CRAFT was not developed with problem gambling in mind, and it relies heavily on the CSO being able to tell when a person is intoxicated. Gambling can be done anywhere and at anytime and is easy to hide. Therefore, reinforcing intermittent abstinence from gambling is often very difficult. Consequently, our approach will focus less on the CSO being able to tell when the IP has gambled and more on creating an environment that encourages gambling-free activities. The aim is for the CSO and IP to engage in naturally reinforcing activities alone and together, thus hopefully reconnecting with each other and reintroducing non-gambling related reinforcers to the IP. The CSO is also introduced to concepts from MI, such as ‘the stages of change’, ‘asking for permission’ and the concept of ‘resistance’. The purpose is to help the CSO find situations where the IP is more open to change, instead of inadvertently creating resistance. Concrete examples are given of different ways to avoid resistance, and how to lead the conversation forward. Concepts from IBCT are also integrated into the programme. For instance, ‘contingency based change’ is one of the purposes of trying to get the CSO and IP to engage in more activities together, that is, reinforcement from spontaneous positive behaviours. IBCT's concept of ‘acceptance’ is also introduced to help the CSO to better understand the IP's learning history and therefore better cope with the situation. There are also several concepts and exercises that focus on CSOs in their own right. The rationale is that problem gambling has led to the CSOs losing important positive reinforcers in their lives. Therefore, there are reoccurring exercises to engage the CSO in reinforcing activities. The CSOs are prompted to schedule and log these activities. A short summary of the individual modules is provided in table 1.

**Table 1:** Programme contents

Module	Summary content
1. Psychoeducation about gambling problems	Information about the programme and technical platform Gambling problems in general, signs of gambling, and the biopsychosocial model Goals, and how the gambling problem started
2. Functional analysis and gambling free activities	Functional analysis with exercises Gambling urges Alternatives to gambling Reinforcing non-gambling behaviour
3. Rewards and behavioural activation for both the CSO and problem gambler	Helping CSOs reconnect with their values Behavioural activation and rewarding themselves Strategies that make the CSO feel worse Reconnecting with the gambler; doing things together
4. Psychoeducation about motivation and protecting the CSO’s economy	CSO's motivation to support the IP Motivation and gambling; ‘stages of change’ How to talk about gambling and avoiding resistance; ‘asking for permission’ Protecting the CSO's economy Lending money and enabling
5. Common behaviours that inadvertently enable gambling	Enabling Natural negative consequences
6. Communication training and principles from MI	Rolling with the punches Effective communication; ‘soft disclosures’ Active listening and reflections
7. Problem-solving	Problem-solving with exercises Interactive log to perform the steps in problem-solving
8. Inviting the gambler into treatment	Identifying when motivation is high Different treatment options Examples of how to use communication skills Support during treatment Relapses
9. Repetition and evaluation	Repetition, evaluation, and creating an action plan

CSO, concerned significant other; IP, identified patient; MI, motivational interviewing.

The programme will be given as guided self-help with guidance given via a secure email system and telephone. There are nine modules, all of which contain homework exercises and about 5–10 pages of text. Every week a new module is made available to the participant, regardless of whether the previous module has been completed. At the start of the study, the participant is informed that the counsellor will be aiding them for a maximum of 10 weeks. After these 10 weeks, the participant will still have access to the modules but not the counsellor.

All CSOs will receive help from their counsellor in locating professional gambling treatment as close to their home as possible. The National Gambling Helpline has a registry of available treatment options in Sweden, which is regularly kept up to date. In parallel to this study, we are also running a trial on internet CBT for people with problem gambling. The CSO's IP who wishes to enter treatment will be offered the programme used in the parallel study.

#### Wait-list condition

The participants allocated to the control condition will be put on a waiting list and offered the treatment after 10 weeks. The participants will know that they have been randomised to the control group. During these 10 weeks, they will participate in the weekly assessments. The CSOs will receive information about available treatment options—in their area and web-based—for problem gambling.

### Outcome measures and data collection

See table 2 for a list of measures and when they will be collected. All outcomes will be self-reported via the internet. The primary outcome concerns gambling behaviour and consequences for the IP and their CSO. This will be measured by the Inventory of Consequences Scale for the Gambler and CSO (ICS).^45^ The scale was adopted from the substance abuse field and consists of three subscales: (1) consequences for the gambler, (2) negative emotional consequences for the CSO and (3) negative behavioural consequences for the CSO. It was used in a similar study with CSOs of people who gamble.^20^ Internal consistency was good, ranging from α=0.86 to 0.89 for the different subdomains. Test-retest reliability was excellent over 7 to 10 days (ICC=0.93 for all domains).^45^ Although lacking an extensive psychometric evaluation, these results indicate good psychometric properties in a relevant sample. Gambling behaviour will be reported by the CSO, and will be measured by the timeline followback method for the past 30 days, and continuously during the study. CSOs will be asked to report days gambling and money spent. Previous studies have found fair agreement between reports from CSOs and the IP,^45^ indicating that the CSO's report of gambling behaviour is reasonably valid and reliable as a proxy measure of problem gambling behaviour. CSOs will also be asked to report whether and when the IP decided to enter treatment. Treatment engagement is defined as completing at least one treatment session or agreeing to call the National Gambling Helpline. We choose to include calls to the Helpline since they work with MI, and research has shown that such brief interventions can reduce gambling problems.^46^

**Table 2:** Outcomes and their placement during the study

Outcome	Measure	Pretest	Weekly during treatment*	Post-test, 6, 12 months
Primary outcome				
Gambling consequences	ICS	X	X	X
Secondary outcomes				
Treatment engagement	–	X	X	X
Gambling behaviour	TLFB: days, money	X	X	X
Depression	PHQ-9	X	X	X
Anxiety	GAD-7	X	X	X
Relationship	RAS	X	X	X
Quality of life	WHOQOL-Bref	X		X

*Not all measures are answered by all participants every week; see the section about ‘planned missingness design’.

GAD-7, Generalised Anxiety Disorder Scale;^48^ ICS, Inventory of Consequences Scale for the Gambler and CSO;^45^ PHQ-9, Patient Health Questionnaire-9;^47^ RAS, Relationship Assessment Scale;^52^ TLFB, Timeline followback method;^68^ WHOQOL-Bref, WHO Quality of Life Questionnaire-BREF.^53^

PHQ-9^47^ and GAD-7^48^ will be used to measure symptoms of depression and anxiety. PHQ-9 contains nine items, and scored 0–3 with a total score between 0 and 27.^49^ GAD-7 is frequently used to assess general anxiety, and contains seven items (scored 0–3). Both PHQ-9 and GAD-7 are well-established measures with demonstrated good validity and reliability even when administered via the internet.^49–51^ Relationship satisfaction will be measured by the generic version of the Relationship Assessment Scale (RAS).^52^ RAS consists of seven items and has shown good psychometric properties with CSOs of problem gamblers.^45^ The short version of the WHO Quality of Life Questionnaire will be used to measure the CSO’s quality of life; it consists of 26 items and has demonstrated good reliability and validity.^53^

### Data monitoring

Since all outcomes are collected online, the risk of data loss or corruption is minimal. The data are stored encrypted and are only accessible by the people running the study. The collection and storage of data will adhere to the Swedish Personal Data Act.^54^ This study will not have a formal Data Monitoring Committee and no interim analysis will be performed. Previous studies and clinical experience indicate minimal risk for the participants. Moreover, participants will be asked about any adverse events experienced during the study period.

### Planned missingness design

The study will utilise a planned missingness design for the weekly measures.^55^ This is to decrease the number of items each participant must answer each week, but still retain a good temporal resolution. Each participant will be randomised to one of two measurement schemes. This design effectively leads to biweekly measures for Inventory of Consequences Scale for the Gambler and CSO (ICS) and weekly measures for PHQ-9, GAD-7, RAS and TLFB. Table 3 outlines the two variants.

**Table 3:** Planned missingness design for the weekly measurements, participants are randomly assigned to one of two measurement schemes

	Days from randomisation								
	0	7	14	21	28	35	42	49	56
Scheme 1	X	O	O	O	X	O	O	O	X
Scheme 2	X	O	X	O	O	O	X	O	O

X=ICS only; O=PHQ-9, GAD-7, RAS and TLFB (last 7 days).

GAD-7, Generalised Anxiety Disorder Scale; ICS, Inventory of Consequences Scale for the Gambler and CSO; PHQ-9, Patient Health Questionnaire-9; RAS, Relationship Assessment Scale; TLFB, Timeline followback method.

### Process measures

To better understand what mechanisms mediate change during the study, data on treatment involvement will be collected, in addition to the weekly measures. Treatment involvement will be measured as data completion, time spent with the treatment site and the number of page views on the site, and will be collected unobtrusively as participants visit the treatment site.

### Planned subgroup contrasts

It is hypothesised that the following factors will predict treatment response: (1) type of relationship with the IP (parent, romantic partner or other) and (2) if the CSO lives with the IP.

### Randomization

CSOs will be randomised to one of the two treatment arms (1:1 ratio) after eligibility and pretest assessment is completed. The allocation sequence will be generated by a computer random number generator. To ensure balanced groups, block randomisation will be used. Each block's size will be randomly chosen from the set (4, 6, 8), and be unknown to the researchers involved in the study. A research assistant who is independent from the study will perform the treatment allocation, using sealed, sequentially numbered, opaque envelopes.

### Statistical analyses

Owing to the hierarchical structure of the data and the planned missingness design, analyses will be performed within the linear mixed models framework, such as to model the variability and dependency at the different levels. Treatment entry rates will be analysed using discrete-time event history models (ie, survival analysis).^56^ Survival analysis enables the evaluation of *whether* and *when* events occur; this will be used to compare time to treatment entry and differences in treatment entry rates in the study. Continuous outcomes will be analysed using a linear mixed models approach.^57^ Model building will follow the data-driven and theoretical approach described in Singer and Willet.^56^ Time will be split into two periods by a piecewise linear function^58^; this makes it possible to parsimoniously model change during treatment and follow-up data. Additionally, we hypothesise that treatment engagement will be associated with a reduction on the ICS self-report, and will test this hypothesis by joint modelling.^59^ Furthermore, for the analysis of the timeline followback reports (count data), it is anticipated that the data will be positively skewed and bounded at zero. Hence, generalised linear mixed models will be fitted, specifically zero-inflated Poisson models. In the case of overdispersion, zero-inflated negative binomial regression models will be fit.^60^

#### Handling of attrition

All randomised CSOs will be included in the statistical analyses, that is, an intention-to-treat analysis will be used.^61^ If the pattern of the non-responses is attributable to observed data, then the attrition is said to be *missing at random (MAR)*. Under the MAR assumption, the maximum likelihood approach and multiple imputation will yield sensible parameter estimates.^62^ Unfortunately, it is impossible to prove that the responses are MAR; consequently, pattern-mixture methods will be used in order to perform sensitivity analyses.^63^

#### Sample size

The study's sample size is based on power calculations for the primary outcome (ICS). Since no good parameter estimates are available for this study, standardised coefficients are used. Power is estimated for the primary between-groups comparison directly post-treatment. A linear mixed model with random intercept and slopes is assumed. First, it is assumed that the between-groups standardised mean difference (Cohen's d) will be at least 0.5 at post-test, standardised using the SD at baseline. Moreover, the individual heterogeneity in change is likely to be large. Therefore, individual change at post-treatment is estimated to have an SD of 0.8 around the standardised average estimate (ie, variance due to random slopes). This amount of heterogeneity means that the 95% prediction interval for individual treatment response is expected to vary between ±1.6 around the average change. Assuming a standardised within groups difference of 1, these estimated numbers imply that about 10% of the participants will be unimproved or have negative outcomes (given by the cumulative distribution function of the Gaussian distribution). Moreover, at post-treatment, we estimate that 75% of the variance will be between participants and a 25% residual variance. A shift in this ratio towards more residual variance will decrease power. Given these estimates, 75 participants are needed per group to achieve approximately 80% power, with α=0.05 (this power calculation used equation 2 in Ard and Edland^64^).

Moreover, on the basis of the treatment entry numbers reported in previous studies,^20^ ^29^ it is estimated that the treatment entry rate for the wait-list group will be 15%. Thus, using formulas to calculate power for a test of two independent proportions,^65^ it is estimated that 75 CSOs per group will achieve 80% power (α=0.05) if the treatment entry rate in the CBT-CSO group is 35%. In this scenario, the power for a test of two proportions and the power for a survival analysis are essentially the same. Hence, power is not reported for a survival analysis.

## Discussion

This study will test the efficacy of a CBT-based programme for CSOs of people with problem gambling. Currently, no support programmes aimed at these CSOs have received enough research support to be considered a ‘well-established’^66^ empirically supported treatment. Since the intervention will be internet-delivered, the potential for wide distribution is evident. This opens the potential to provide assistance to all CSOs in Sweden, especially to the majority of CSOs who live in cities without the existence of any peer-support groups or professional help. Thus, the development and evaluation of internet-based assistance for these CSOs is deemed to be exceptionally important. Moreover, the implications of potentially getting treatment-refusing individuals to seek gambling treatment earlier cannot be overstated. Our prediction is that the present study will improve our knowledge of how to get people with problem gambling to enter treatment, reduce their harmful gambling behaviour, and help their CSOs cope with the gambling, thus hopefully improving the quality of life of the people who gamble, the CSOs, and reducing the impact of problem gambling on the community at large. Moreover, no studies have been conducted with this population in Sweden. This study will therefore provide important information on the feasibility of providing internet-based support to CSOs of treatment-refusing people with problem gambling.

## Limitations

There are several potential limitations to this design. First, there is only limited research done on the main outcome measure, and how well CSOs provide valid reports of gambling behaviour. Moreover, the feasibility of this type of intervention is unknown. Therefore, adherence to the programme and attrition from the study are potential challenges. Lastly, the wait-list design will not enable between-group comparison for long-term follow-up measures. Thus, it will not be possible to know how the programme affects relapse rates in the long term. Despite these limitations, this study will hopefully provide preliminary evidence regarding the feasibility and efficacy of the programme.

## Ethics and dissemination

The protocol has been approved by the regional ethics board of Stockholm, Sweden. Written informed consent will be obtained via mail from all participants, and all participants will be informed that they can withdraw from the trial at any time.

The results of this trial will be submitted for publication in peer-reviewed journals, no matter the results. Findings will also be disseminated at gambling conferences aimed at researchers and practitioners. Moreover, after the study is completed, it is possible for an institution like the Helpline to incorporate the CBT-CSO method in their regular operations.

In the spirit of open science, an anonymised version of the data set generated in this trial will be published in a data repository (eg, Dryad or figshare), accompanied by script files to reproduce the statistical analyses. In addition to the CONSORT statement, the guidelines for executing and reporting internet intervention research will be adhered to.^67^
